# Books and Magazines

**Published:** 1911-10

**Authors:** 


### Books and Magazines.

---

THE PRACTICAL MEDICINE SERIES.—Comprising Ten Volumes on the Year's Progress in Medicine and Surgery. Under the general editorial charge of Gustavus P. Head, M. D., Professor of Laryngology and Rhinology, Chicago Post-Graduate Medical School; Charles L. Mix, A. M., M. D., Professor of Physical Diagnosis in the Northwestern University Medical School. Volume IV—Gynecology. Edited by Emilius C. Dudley, A. M., M. D., Professor of Gynecology, Northwestern University Medical School; Gynecologist to St. Luke's and Wesley Hospitals, Chicago, and C. von Bachellé, M. S., M. D., Assistant Professor of Obstetrics, Chicago Policlinic and College of Physicians and Surgeons; Gynecologist to the German Hospital, Chicago. Series 1911. Chicago: The Year-Book Publishers, 180 North Dearborn Street.

Volume IV, Gynecology, and Volume V, Obstetrics, are now ready for delivery. Volumes I, II and III have been noticed heretofore. This series is so well known and so popular that I deem it superfluous to say anything in commendation of it. It keeps the reader abreast of the times, being cullings from the journals, and contains all that is new and valuable; matter which will later be incorporated in text-books. It is the advance guard in the march of progress.

Please note that the present volume is one of a series of ten issued at about monthly intervals, and covering the entire field of medicine and surgery. Each volume being complete for the year prior to its publication on the subject of which it treats.

Price of these volumes is $1.25 each. Price of the series of ten volumes, $10.

We desire to call attention to the fact that this series is published primarily for the general practitioner, at the same time the arrangement in several volumes enables those interested in special subjects to buy only the parts they desire.

---

MANUAL OF THE DISEASES OF THE EYE.—For Students and General Practitioners. By Charles H. May, M. D., Chief of Clinics and Instructor in Ophthalmology, College of Physicians and Surgeons, Medical Department, Columbia University, New York, 1890-1903; Ophthalmic Surgeon Mt. Sinai Hospital, New York, etc. Seventh edition, revised, with 362 original illustrations, including 22 plates, with 62 colored figures. New York, 1911. Wm. Wood & Co. Price, $2.00, net.

May's Manual has been a favorite many years. It has frequently been reviewed in this JOURNAL, and needs nothing more to be added, except that it is revised and much improved in get-up.

---

ONE HUNDRED SURGICAL PROBLEMS.—The Experiences of Daily Practice Dissected and Explained. By James G. Mumford, M. D., Visiting Surgeon to the Massachusetts General Hospital; Instructor in Surgery, Harvard Medical School, etc. 8vo, 354 pages. Price, $3.00. Boston: W. M. Leonard, Publisher, 1911.

Here is a book of clinical histories in which we see, not detached groups of symptoms, but actual patients and real persons.

It presents one hundred selected cases, well classified in groups to show the phases of each subject, with statement of symptoms and thorough discussion leading to diagnosis, treatment and results.

Of the value of this kind of book there is no question. As the preface states: "The method in proper hands is luminous and the lessons instantly comprehensible."

Certain groups of cases, as those of the stomach and duodenum, Graves' disease, digestive orders are worthy of special mention. In the last named series are tell full-page X-ray plates showing ptosis of the large intestine. These are equally valuable to the internist and the surgeon, and superior to any work published up to the present in this field.

Each case history has been thoughtfully considered and well-written,—in accord with a style which Dr. Mumford has made his own. The book comprises a valuable post-graduate course in surgery presented in most interesting form.

GOLDEN RULES OF PEDIATRICS.—Aphorisms, Observations, and Precepts on the Science and Art of Pediatrics. Giving Practical Rules for Diagnosis and Prognosis, the Essentials of Infant Feeding, and the Principles of Scientific Treatment. By John Zahorsky, A. B., M. D., Clinical Professor of Pediatrics, Medical Department Washington University, St. Louis; ex-President of the St. Louis Pediatric Society; Attending Physician to the Bethesda Foundlings' Home and the St. Louis Children's Hospital; Member of the American Medical Association and St. Louis Academy of Science; Author of "Baby Incubators," etc. With an introduction by E. W. Saunders, M. D., Emeritus Professor of Diseases of Children and Clinical Midwifery, Medical Department Washington University, St. Louis, etc. Price, $2.50. St. Louis: C. V. Mosby Company, 1911.

The nature, objects and purposes of this book are sufficiently set forth above, and it needs no comments.

---

A HAND-BOOK FOR MEDICAL ADVERTISERS.—Compiled and published by Henry R. Harrower, M. D., Medical Advertising, 921-31 Schiller Building, Chicago, Illinois. 1911.

A comprehensive little book of ready reference, giving data regarding the medical, drug dental and allied journals published in the United States and adjacent countries, together with other information of interest to medical and drug advertisers.

One Thousand Surgical Suggestions.—By Walter W. Brickner, B. S., M. D., Adjunct Surgeon Mt. Sinai Hospital; Editor in Chief American Journal of Surgery, with the collaboration of James P. Warbasse, M. D., Harold Hays, M. D.; Eli Moschcowitz, M. D., and Harold Neuhof, M. D. 225 pages. Cloth bound, semi-de Luxe, $1.00. Full de Luxe leather, $2.25. Surgery Publishing Company, 92 William Street, New York, U. S. A.

This is one of the biggest little books ever presented to the profession. In its 225 pages are found a collection of 1000 epigrammatic succinct, virile and instructive hints based upon actual experience and everyone a lesson in itself.

The suggestions are so arranged and indexed that all subjects covered can be immediately referred to and the particular hint upon any particular subject immediately found. It bristles with pointed and useful suggestions which in many cases might just turn the scale from failure to success. Its mechanical presentation is a feature worthy of mention. It is square cloth bound stamped in gold, printed upon India tint paper with Cheltenham type with special marginal side headings in red. A dollar could not be better invested than in the purchase of this book.

---

LITORIA ALIENA.—By *Medicus Peregrinus.* Published by W. M. Leonard, Boston, 1911. Price, 50 cents.

This is a series of letters sent to the *Boston Medical and Surgical Journal* by one of its editors during a recent European trip. These letters present scenes and interests as they appear and appeal to a physician, and contain much of that rarity in medical literature—reading that is both entertaining and instructive.

---

HIERONYMUS FRACASTOR'S SYPHILIS, FROM THE ORIGINAL LATIN. —A Translation in prose of Fracastor's immortal poem. Printed on hand-made imported paper; library binding. Crown octavo. The Philmar Company, Medical Publishers, Fidelity Building, St. Louis, Mo. Price, $2.00.

Here is a remarkable book; as curious as it is interesting. It is excellent reading: as smooth as silk. To those of our readers who have a fondness for the classic in literature it will be a source of much delight.

Syphilis, the author says, originated with one Syphilus—a shepherd who attended the flocks of a great king named Alcithous, whose domain was evidently somewhere on or about the Orinoco in South America, and the author evidently means Columbus when he speaks of the Spaniards who went amongst the natives and there acquired the disease and carried it to Europe. In another place he says the disease, which he attributes to the malign influence of the stars, is an atmospheric epidemic, attacking lots of people simultaneously (without a hint anywhere in the book that it is a sexual disease); was carried by the Gauls (French) into Latium (ancient, very ancient Rome) in their wars. This was long anterior to the time of Columbus, or of the Spanish invasion by Charles VIII, and he calls the disease "Syphilis *sive* Morbus Gallicus." He thus leaves us in ignorance really of the time or place where it originated.

The remedy, he says, in one place is guiac, the sacred tree of the West Indies and South America, where, he says, it was found by the Spaniards, whose first landing was San Salvador, and whose second was up the Orinoco, a country the author calls "Ophir," and he gives a very fanciful account of the plant and its wonderful virtues in curing this new plague. Elsewhere he extols mercury as the remedy, and gives a still more fanciful account of getting mercury out of a cavern in the bowels of the earth by one Ilceus, who had contracted the disease; sent on him by Diana because the Spaniards had killed a bird sacred to her; Ilceus being guided by a presiding divinity, a nymph named Callirhoe. It reads like the "Arabian Nights."

I reproduce Fracastor's formula for using mercury. He says (p. 41): "I prefer to alloy it with a mixture of black hellebore, orris root, galbanum, asafoetida, oil of mastic and oil of native sulphur. Patience! A truce to the disgust, which may be caused by this remedy! For, if it is disgusting, the disease is still more so. Besides, your cure is at this price. So, without hesitation, spread this mixture on your body, and cover with it your entire skin, with the exception of the head and of the precordial region. Then carefully wrap yourself in wool and tow; then get into bed, load yourself with bed covering, and thus await until a sweat bathes your limbs with an impure dew. Ten days in succession renew this treatment,—for ten entire days you are to undergo this cruel trial, whose beneficial effect will not cause you to wait. * * * As a matter of fact, very soon an infallible presage will bring to you the hour of your freedom; very soon you will feel the ferments of the disease dissolve themselves in your mouth in a disgusting flow of saliva,—and you will see the virus, even the virus, evacuate itself at your feet in rivers of saliva."

Verily, there is nothing new under the sun.

Hieronymus Fracastor was an Italian physician, born in 1483, died in 1553. This poem, his masterpiece, was published in 1530. He was professor of logic at Padua.

---

VACCINE THERAPY IN GENERAL PRACTICE.—By Geo. H. Sherman, M. D., Detroit, 1911. Published for the author. Price, not stated.

The author says:

"Rest assured that the subject of vaccine therapy in general practice is worthy of far greater study by the masses of the profession than it has as yet received, and not only will the time taken to acquaint yourself with this work make you a more efficient physician, but it will inevitably add to your bank account—by no means a disadvantage!"

---

HAND-BOOK OF SUGGESTIVE THERAPEUTICS, APPLIED HYPNOTISM, PSYCHIC SCIENCE.—A Manual of Practical Psycho-therapy, designed especially for the general practitioner of medicine and surgery. By Henry S. Munro, M. D., Omaha, Neb. Third edition, revised and enlarged. St. Louis: C. V. Mosby Co., 1911. Price, $4.00.

This third edition of the work contains eight entirely new chapters, which, together with the enlarged and rewritten chapters of the preceding edition, constitute an embodiment of the recent advances in psychotherapy.

Now here is a book that is really worth while. It is intensely, absorbingly interesting—and instructive. It is the teaching and illustrations of a pioneer in a field unexplored, and as full of mystery as of fact, who tells of his experiences in the new art of psycho-therapy. Ultimately, all healing of disease will be through mental influences—when we learn how to administer the remedy, and drugs—*per se*—will be relegated to the attic of worn-out things. (That is my opinion, not Dr. Munro's.) He says he is not a therapeutic nihilist. I am, very nearly so, and have long regarded a bottle of medicine as a kind of fetich, its curative properties being, in many cases, in direct proportion to the patient's faith in his doctor, or the drugs. Beyond doubt, in the application of suggestion, we have a remedy of great potency—applicable to a wide range of diseases,—a remedy which can be used in time by every physician, with great *eclat,* when he shall have learned more about it. Dr. Munro has blazed the way, and his splendid book, written in a style as fascinating as the subject itself, is a beacon light for us to follow. I earnestly commend it to my readers. They will get as much enjoyment out of it as I did.

Like evolution, hypnotism (unfortunate name!) the manifestation of a powerful, and, as yet, mysterious force, is no new thing; it has always existed. It is man who just now begins to see and recognize the force, and attempts to analyze and use it. My own opinion is that thought—indeed, all mental processes—is electrical, and the brain is the original dynamo, which transforms the energy we get from food, air and sun, into electrical energy, which is expressed in all our senses and functions—sight, hearing, feeling, action and being, as well as the functions of the internal organs. See "Strange Case of Dr. Bruno."

---

PLAIN, PRACTICAL, MEDICAL AND HYGIENIC TALKS FOR THE FAMILY CIRCLE.—By Chester W. Harper, M. D., Oph. D., N. D., Kingfisher, Okla., Vice-President of the Oklahoma State Association for the Prevention and Cure of Tuberculosis. Price, 50 cents, postpaid. 1910.

We do not approve of "Family Doctors" as a rule, but the teaching of this little book on the subject of hygiene is commended. Every family should know about it. I see the doctor recommends "a bath once a week." My! this hot weather,—one feels like living in a bath tub.

